# Decrosslinking enables visualization of RNA-guided endonuclease–*in situ* labeling signals for DNA sequences in plant tissues

**DOI:** 10.1093/jxb/erz534

**Published:** 2019-11-30

**Authors:** K Nagaki, N Yamaji

**Affiliations:** 1 Institute of Plant Science and Resources, Okayama University, Kurashiki, Japan; 2 Trinity College Dublin, Ireland

**Keywords:** Centromere, CRISPR/Cas9, epigenetic modifications, immunohistochemistry, *in situ* DNA visualization, RNA-guided endonuclease–in situ labeling (RGEN-ISL), telomere

## Abstract

Information about the positioning of individual loci in the nucleus and the status of epigenetic modifications at these loci in each cell contained in plant tissue increases our understanding of how cells in a tissue coordinate gene expression. To obtain such information, a less damaging method of visualizing DNA in tissue that can be used with immunohistochemistry is required. Recently, a less damaging DNA visualization method using the CRISPR/Cas9 (clustered regularly interspaced short palindromic repeats/associated caspase 9) system, named RNA-guided endonuclease–*in situ* labeling (RGEN-ISL), was reported. This system made it possible to visualize a target DNA locus in a nucleus fixed on a glass slide with a set of simple operations, but it could not be applied to cells in plant tissues. In this work, we have developed a modified RGEN-ISL method with decrosslinking that made it possible to simultaneously detect the DNA loci and immunohistochemistry signals, including histone modification, in various types of plant tissues and species.

## Teaser

A simple CRISPR/Cas9-based method, RNA-guided endonuclease–*in situ* labeling, made it possible to detect specific DNA sequences in tissues of diverse plant species.

## Introduction

Chromatin in eukaryotic cells is highly organized to minimize the volume of genomic DNA and to control various functions of the genome, including gene expression, replication, recombination, and DNA repair (Hübner *et al.*, 2013). The organized chromatin in the nucleus and specific positions of distinct chromatin regions are closely related to their functions. In particular, for cell-type-specific regulations, each cell should have specific positioning and modifications of the chromatin.

Fluorescent *in situ* hybridization (FISH) has been used to visualize specific DNA sequences in the nucleus. Although the FISH method is versatile and can be applied to various species and cell types, it involves a denaturing process to make the DNA single-stranded. The denaturation and fixatives used for FISH cause structural changes in the samples, and the changes become a barrier to three-dimensional (3D) DNA locus analyses (Kozubek *et al.*, 2000). Thus, there is a demand for developing non-denaturing DNA visualization methods for 3D analyses.

First, combinations of an operator and repressor fused with fluorescent protein (LacO/LacI or TetO/TetR) were employed as non-denaturing methods of DNA visualization, and the same methods were used for live imaging in Arabidopsis (Kato and Lam, 2001; Matzke *et al.*, 2005). However, these methods required the insertion of the operator sequence into the desired locus by transformation to visualize the specific locus. Next, non-denaturing DNA visualization methods to detect native DNA sequences were developed using genome editing systems, including zinc finger (Lindhout *et al.* 2007), transcription activator-like effectors (Ma *et al.*, 2013; Fujimoto *et al.*, 2016), and clustered regularly interspaced short palindromic repeats (CRISPR)-CRISPR-associated caspase 9 (Cas9) systems (Chen *et al.*, 2013; Dreissig *et al.*, 2017). Although these systems do not require transformation of the operator sequences, they require the intracellular expression of proteins for genomic editing fused to fluorescent proteins. More recently, DNA visualization methods using extracellular Cas9 were reported, and these methods could be applied to fixed cells (Deng *et al.*, 2015; Ishii *et al.*, 2019). In particular, one of these approaches, RNA-guided endonuclease–*in situ* labeling (RGEN-ISL; Ishii *et al.*, 2019), is a simple and fast method that has been used to detect repetitive DNA sequences in fixed plant and human cells and is compatible with immunostaining. The method was used to visualize target repetitive DNA sequences by combining target-sequence-specific CRISPR RNAs (crRNA) with a universal labeled *trans*-activating crRNA (tracrRNA). Theoretically, the combination of this method and immunostaining with anti-modified histone antibodies would make it possible to analyze the epigenetic status of the target chromosomal regions. This method has the potential to be used for observation of these statuses in plant tissues. However, RGEN-ISL was applied only to isolated nuclei in the work described by Ishii *et al.* (2019), and no results of RGEN-ISL in plant tissue have yet been reported. Therefore, the applicability of this method to cells in tissue is unknown.

In this research, we applied the RGEN-ISL method for DNA visualization in plant tissues. Since the original RGEN-ISL method was not able to visualize target DNA sequences in plant tissues, we attempted to modify the method to achieve this goal. The resulting RGEN-ISL method with defixation allowed the visualization of target DNA sequences in plant tissues, and the modified method was applicable to many plant species and tissues. Since this method is also compatible with immunohistochemistry, we believe it will be a powerful method for the high-resolution analysis of DNA–protein coexistence in cells in plant tissues.

## Materials and methods

### Plant material

Sunflower (*Helianthus annuus*, 2*n*=2*x*=34), soybean (*Glycine max*, 2*n*=2*x*=40), common bean (*Phaseolus vulgaris*, 2*n*=2*x*=22), and tomato (*Solanum lycopersicum* cv. Micro-Tom, 2*n*=2*x*=24) were germinated and grown in pots with soil. Tomato seeds (TOMJPF00001) were provided by the University of Tsukuba Gene Research Center through the National Bio-Resource Project of the Ministry of Education, Culture, Sports, Science and Technology, Japan. Roots and stems were collected from seedlings of each species 3 days after germination. Tissue samples were obtained from the leaves of 1-week-old plants and the petals of 1-month-old plants using a paper puncher with 5 mm diameter cutters.

Seeds of *Arabidopsis thaliana* ecotype Col-0 (2*n*=2*x*=10) and tobacco (*Nicotiana tabacum* L. SR1, 2*n*=4*x*=48) were sterilized with 70% ethanol and germinated on Murashige and Skoog medium. The tobacco seeds were a gift from Japan Tobacco Inc. One-week-old seedlings were collected.

Rice (*Oryza sativa* cv. Nipponbare, 2*n*=2*x*=24) seeds were germinated and grown on a net floated on 0.5 mM CaCl_2_ solution in a plastic container; 4-day-old roots were collected.

### Fixation

For fixation, collected roots and seedlings were soaked in fixatives in microtubule-stabilizing buffer (5 mM PIPES, pH 6.9, 0.5 mM MgSO_4_, and 0.5 mM EGTA) containing 1.5% or 3.0% (w/v) paraformaldehyde (PFA) and 0.3% (v/v) Triton X-100. Penetration of the fixative into the tissues was achieved by subjecting the samples to three cycles of vacuum (–50 kPa) for 1.5 min each with release at room temperature. After fixation, the tissues were washed twice in phosphate-buffered saline (PBS) for 10 min at room temperature.

Fixation, permeabilization, and clearing were performed consecutively for the leaves, stems, and petals using a modified ePro-ClearSee method (Kurihara *et al.*, 2015; Nagaki *et al.*, 2017). First, the samples were fixed in fixative containing 3.0% PFA and washed in PBS as described above. Next, the fixed samples were soaked in a mixture of 0.2% (w/v) cellulase Onozuka RS (Yakult Pharmaceutical Industry, Tokyo, Japan) and 0.1% (w/v) pectolyase Y-23 (Seishin Pharmaceuticals, Tokyo, Japan) dissolved in PBS with 0.3% (v/v) Triton X-100, and digested by incubation for 60 min at 37 °C. Then, the samples were washed twice in PBS for 10 min each at room temperature. The samples were permeabilized by soaking in 2-propanol for 10 min and cleared in ClearSee solution [10% (w/v) xylitol, 15% (w/v) sodium deoxycholate, and 25% (w/v) urea in water] at room temperature for 7 days. The ClearSee solution was changed once daily. After the tissues had been cleared, they were rinsed once with 50% (v/v) 2-propanol in water for 10 min at room temperature and then washed twice in PBS for 10 min at room temperature.

### Preparation of nuclei

Nuclei of tomato leaves were spread on glass slides for a pilot experiment to determine the optimum temperature for the RGEN-ISL method. The 3.0% PFA-fixed and washed tomato leaves were digested with a mixture of 1.0% (w/v) cellulase Onozuka RS and 0.5% (w/v) pectolyase Y-23 dissolved in PBS, by incubation for 60 min at 37 °C. Nuclei released by the digestion were compressed on to slides coated with poly-l-lysine (Matsunami, Osaka, Japan).

### Sectioning

The fixed roots of the sunflower, soybean, common bean, and tomato plants and the cleared sunflower stems were directly glued to the stage of a microslicer (LinearSlicer PRO10; Dosaka EM, Kyoto, Japan) with instant adhesive and then sectioned at 100 μm thickness. Samples of the rice roots and seedlings of Arabidopsis and tobacco were embedded in 5% agar and then sectioned as described above.

### Purification of HaloTag-fused SpdCas9

A HaloTag-fused *Streptococcus pyogenes* Cas9 protein containing the double nuclease mutation (D10A and H840A; dCas9-Halo) was expressed in *Escherichia coli* strain BL21 (DE3) using the pET302-6His-dCas9-Halo plasmid (Deng *et al.*, 2015; Addgene plasmid no. 72269). After induction with 1 mM isopropyl β-d-1-thiogalactopyranoside, the 6His-dCas9-Halo proteins were purified using TALON Single Step Columns (Clontech, Palo Alto, CA, USA) following the manufacturer’s instructions. The purified proteins were concentrated using Amicon Ultra 100 K filters (Merck Millipore, Burlington, MA, USA) and were exchanged into a storage buffer [50 mM HEPES (pH 7.5), 150 mM KCl, 1 mM Tris(2-carboxyethyl)phosphine, 20% (v/v) glycerol]. The dCas9 proteins were stored at –20 °C.

### Formation of ribonucleoprotein

The ribonucleoprotein (RNP) was formed using the Alt‐R CRISPR‐Cas9 system [ (Integrated DNA Technologies (IDT), Coralville, IA, USA] as described in Ishii *et al.* (2019). In brief, two reported (telomeric and centromeric DNA of Arabidopsis; Ishii *et al.*, 2019) and three newly designed crRNAs were used in this research (see Supplementary Table S1 at *JXB* online). The new crRNAs were designed using the CRISPRdirect website (http://crispr.dbcls.jp; Naito *et al.*, 2015) based on reported centromeric DNA sequences of rice (CentO; Cheng, *et al.*, 2002), soybean (G71 clone of GmCent-1; Tek *et al.*, 2010), and common bean (CentPv; Iwata *et al.*, 2013). The crRNAs in Supplementary Table S1 were synthesized by IDT, and guide RNA was formed using the synthesized crRNA and ATTO 550 labeled tracrRNA (IDT) in nuclease‐free duplex buffer [30 mM HEPES (pH 7.5), 100 mM CH_3_COOK]. Then, the guide RNAs were mixed with 6His-dCas9-Halo in Cas9 reaction buffer [20 mM HEPES (pH 7.5), 0.1 M KCl, 5 mM MgCl_2_, 5% (v/v) glycerol, 1% (w/v) bovine serum albumin and 0.1% Tween 20, and 1 mM dithiothreitol] and incubated at 26 °C for 10–15 min to form RNP.

### Decrosslinking

For decrosslinking, the 3.0% PFA-fixed tissue samples were soaked in 20 mM Tris–HCl (pH 9.0) and then incubated for 2 h at 60 °C. The decrosslinked samples were washed twice in PBS at room temperature and the samples were then used for RGEN-ISL.

### RGEN-ISL

The plant tissues were transferred into 150 μl of Cas9 reaction buffer placed on microscope slides containing hydrophilic circular windows with a diameter of 15 mm surrounded by a framework of printed hydrophobic black ink (TF0215; Matsunami, Osaka, Japan) and maintained at room temperature for 5 min for blocking. Then, the same volume of Cas9 reaction buffer containing RNP was added to the slide and mixed by pipetting several times. The slides were placed in a moisture chamber and incubated at 26, 37, or 42 °C for 3–5 h or at 4 °C for 16 h. After the incubation, the reaction was stopped by removing the buffer, and the tissues were washed twice in PBS for 10 min at room temperature. To prevent dissociation of the RNP complex, the tissues were post-fixed with 3.0% PFA for 5–10 min at room temperature, and then washed twice in PBS for 5 min at room temperature. The tissues were mounted with an anti-fade mountant, SlowFade Diamond containing 1 μg/ml DAPI (Thermo Fisher Scientific, Waltham, MA, USA). The slides were placed at 4 °C for at least 8 h until the anti-fading solution had penetrated completely into the tissues.

### Immunohistochemical analysis

To detect kinetochore proteins or histone modifications, immunohistochemistry was conducted after RGEN-ISL. The primary antibody solutions consisted of primary antibody solutions diluted 1:100 in a blocking solution [100 mM Tris–HCl, 150 mM NaCl, and 0.5% (w/v) blocking reagent (Sigma-Aldrich, St. Louis, MO, USA)]: anti-OsCENH3 rabbit antibody (Nagaki *et al.*, 2004) for centromere-specific histone H3 variant (CENH3) in rice or anti-GmCENH3 rabbit antibody (Tek *et al.*, 2010) for CENH3 in soybean and common bean, and anti-H3K9me2 mouse antibody (MABI0317; MBL, Nagoya, Japan). After post-fixing and washing with PBS, the tissues were incubated in the primary antibody solutions at 4 °C for 12–16 h and then washed twice in PBS for 10 min at room temperature. The secondary antibodies used were Alexa Fluor 647-labeled anti-rabbit antibodies (Molecular Probes, Eugene, OR, USA) and Alexa Fluor 488-labeled anti-mouse antibodies (Molecular Probes). Both antibodies were diluted 1:500 with the blocking solution, reacted at 37 °C for 1 h, and then washed in the same way as for the primary antibodies. The tissues were transferred on to glass slides and mounted as above.

### Microscopy

For observation of large areas within the tissue sections, RGEN-ISL signals were observed with a fluorescence microscope (Keyence, Osaka, Japan; BZ-9000) using Nikon CFI Plan Apochromat ×40 and ×60 objectives. Images were captured using Multicolor image capturing software built into the BZ-9000 system, and the captured images were overlaid or joined using the BZ-II Image Analysis Application built into the system. For counting, Z-stack images were captured at 1 μm intervals and RGEN-ISL signals were counted using these images and ImageJ software. Full focus processing was performed by the built-in software to display all signals in a set of the Z-stack images to a two-dimensional image.

To observe the detailed subcellular localization of the RGEN-ISL signals, confocal microscopic analyses were performed on a Leica TCS SP8 X (Leica Microsystems, Wetzlar, Germany) equipped with a 405 nm diode laser and a white light laser (WLL). The excitation and detection wavelengths (nm) were, respectively, as follows: for DAPI, 405 and 440–460; for Alexa Fluor 488, 500 and 508–525; for ATTO 550, 555 and 562–580; and for Alexa Fluor 647, 652 and 658–685. To avoid crosstalk between fluorescence dyes, sequential scanning between the 405 nm diode laser and WLL and time-gated detection for WLL excitation were used. For high-resolution imaging, confocal scanning was performed with a 0.6 Airy unit pinhole size, 43–50 nm xy-pixel size, and 130 nm z-steps. To improve contrast and resolution, confocal raw images were deconvolved by using the Huygens software with default settings.

## Results and discussion

### Determination of the optimum temperature for RGEN-ISL

It has been reported that the RGEN-ISL method can be used at various temperatures (4–37 °C; Ishii *et al.*, 2019). On the other hand, it has been reported that the optimum temperature of Cas9 is 39 °C or higher, and in/del induction rates were extremely reduced at lower temperatures (Xiang *et al.*, 2017). In order to determine the optimum temperature for RGEN-ISL, a pilot experiment was conducted using tomato leaf-derived nuclei spread on slides. In this experiment, RGEN-ISL telomere signals could be visualized at all temperatures tested (4, 26, 37, 39, and 42 °C), but the number of detectable signals and the background levels differed (Supplementary Fig. S1A–E). Less than half of 48 tomato telomeres were detected in the reaction conducted at 4 °C (Supplementary Fig. S1A). More than 40 telomere signals were observed at all the other temperatures, and the background levels decreased with increasing reaction temperature (Supplementary Fig. S1B–E).

### Conditions for RGEN-ISL of plant tissues

**Fig. 1.**
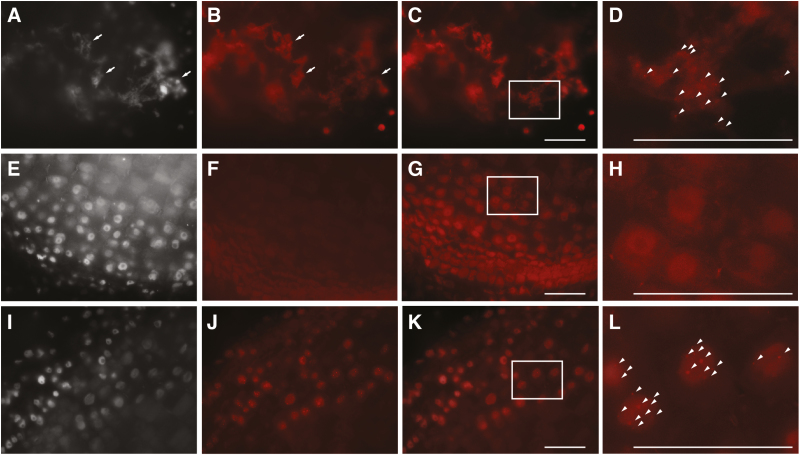
Comparison of fixation and decrosslinking conditions for 3D RGEN-ISL. RGEN-ISL telomere signals in sunflower roots. (A–D) Root section fixed with 1.5% PFA; (E–H) root section fixed with 3% PFA; (I–L) root section fixed with 3% PFA before sectioning and decrosslinked after sectioning. (A, E, I) DAPI-stained nuclei. (B, F, J) RGEN-ISL signals. (C) Merged image of A and B; (G) merged image of E and F; (K) merged image of and I and J. Panels D, H, and L show enlarged images of the areas indicated by boxes in C, G, and K, respectively. Arrows indicate cloud-like signals of damaged nuclei; arrowheads indicate RGEN-ISL signals. Scale bar=50 μm.

To detect RGEN-ISL signals from plant tissues, we first assessed different fixation conditions, using the reported crRNA for telomere sequence, sunflower roots as the tissue, and 42 °C as the reaction temperature. First, we used 1.5% PFA fixation, which is comparable to the reported 4% formaldehyde solution (Ishii *et al.*, 2019). In the fixation, nuclei located at the cut surfaces were physically destroyed and did not retain their original form (cloud-like signals, indicated by arrows in Fig. 1A, B), and RGEN-ISL signals (dot signals, indicated by arrowheads in Fig. 1D) were found in the damaged nuclei (Fig. 1A–D). In addition, non-specific RNP localization was observed in the cloud-like nuclei (Fig. 1C, D). This result suggested that the fixation was too weak to maintain the structure of the nuclei at the cut surfaces.

Next, 3.0% PFA fixation, which is a standard fixation used for immunohistochemical analyses of sunflower roots (Nagaki et al., 2015), was used (Fig. 1E–H). In this case, the nuclei located at the cutting surfaces were cut without structural damage, but RGEN-ISL signals were not observed in the tissues. This result suggested that the fixation was strong enough to maintain the structure of the nuclei but too strong to enable the detection of RGEN-ISL signals. In fact, the RGEN-ISL system is sensitive to fixation and showed the strongest signals in a case without fixation (Ishii *et al.*, 2019).

We considered that loss of the signals might be due to crosslinking of DNA duplexes, and so we tested decrosslinking after sectioning. Theoretically, this method can preserve the structure after sectioning with 3.0% PFA fixation, and genomic DNA can be made accessible to RGEN-ISL by decrosslinking after sectioning. Decrosslinking by heating in Tris buffer at 60 °C for 2 h was conducted. After decrosslinking, RGEN-ISL signals were observed on non-damaged nuclei at the cut surface (Fig. 1I–L). Since the decrosslinking provided the best result, we employed this method as a standard protocol for 3D RGEN-ISL.

The effect of the reaction temperature in 3D RGEN-ISL was confirmed using sunflower root slices and the crRNA for telomeres. Clear telomere signals were observed in the RGEN-ISL reaction conducted at 42 °C (Supplementary Fig. S1I), but weaker signals were observed at 37 °C (Supplementary Fig. S1H), and no signal was observed in the reactions conducted at 4 °C or 26 °C (Supplementary Fig. S1F, G). This result suggests that 3D RGEN-ISL requires a higher activity of Cas9 than two-dimensional RGEN-ISL. Based on these results, all subsequent 3D RGEN-ISL reactions were performed at 42 °C.

To confirm the detection sensitivity of 3D RGEN-ISL, the telomeric signals in different root regions and cells were counted. In the best cases, 58 (a meristem cell) and 59 (a cell in the cortex of the elongation region) of 68 sunflower telomere signals were observed; the median number of signals was 49.5 (*n*=10) in meristem cells and 51.5 (*n*=10) in cortex cells (Supplementary Figs S2A, S3A, B).

### Application of 3D RGEN-ISL

**Fig. 2.**
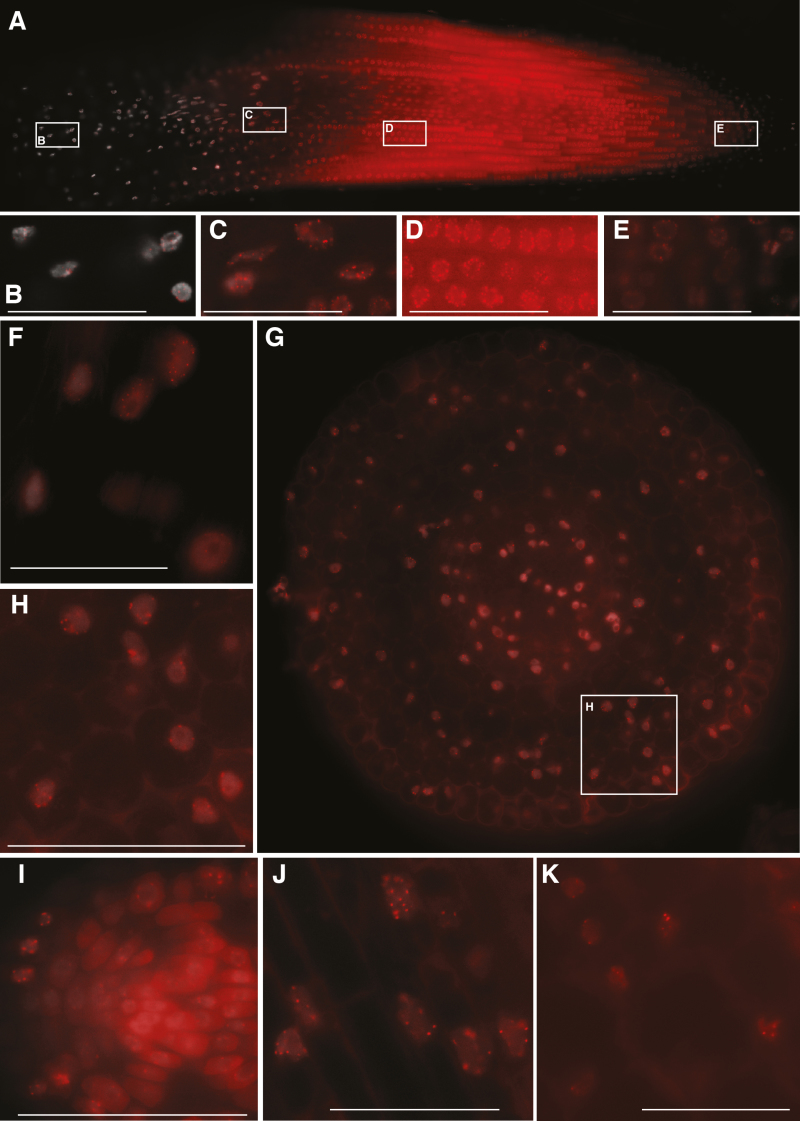
3D RGEN-ISL of root sections. (A–E) RGEN-ISL telomere signals in a tomato root. Panels B–E show enlarged images of the areas indicated by boxes in A. (F) RGEN-ISL telomere signals in an elongation region of tobacco root. (G) RGEN-ISL centromeric signals (CentO) in an elongation region of rice root. (H) Enlarged images of the area indicated by a box in G. (I–J) RGEN-ISL centromeric signals in a root tip of Arabidopsis (180-bp family) (I), an elongation region of soybean root (GmCent-1) (J), and an elongation region of common bean root (CentPv) (K). Scale bar=50 μm.

We applied the 3D RGEN-ISL method to other species and sequences. Clear RGEN-ISL telomere signals were observed using the same crRNA as for the sunflower experiments in tomato and tobacco roots (Fig. 2A–F). In the case of tomato, almost all cells in the 1.4 mm long root slice showed clear RGEN-ISL telomere signals (Fig. 2A–E). In the best cases, 43 of 48 tomato telomere signals were observed in two meristem cells and a cell in the cortex of the elongation region. The median numbers of the signals were 38.5 (*n*=10) in meristem cells and 38.0 (*n*=10) in cortex cells (Supplementary Figs S2B, S3C, D). The sizes of the telomere regions are predicted to be smaller than 10 kb in tomato (Ganal *et al.*, 1991), meaning that the telomere regions contains fewer than 1500 copies of 7-bp telomeric repeats. The crRNA for telomere repeat contains three copies of the telomeric repeat so, theoretically, no more than 500 RNPs can be localized in the 10 kb telomeric regions of tomato. In the 3D RGEN-ISL experiment, the signals were observed in tomato telomere regions (Supplementary Fig. S2B), so 3D RGEN-ISL has the potential to visualize signals from fewer than 500 RNPs localized on a locus. Further, 89 of 96 tobacco telomere signals were observed in two of the cells in the cortex of the elongation region; the median number of signals was 79.5 (Supplementary Figs S2C, S3E).

Additionally, centromeric DNA sequences were detected in the roots of rice, Arabidopsis, soybean, and common bean (Fig. 2G–K). These results imply that the 3D RGEN-ISL method is a universal method for detecting RGEN-ISL signals in root slices of various plant species. In rice, RGEN-ISL signals were observed in meristem cells (median=16.0, *n*=10), cells in the cortex of the elongation region (median=16.0, *n*=10), and cells in the epidermis of the elongation region (median=14.0, *n*=10) by using a crRNA for centromeric tandem repeat (CentO; Supplementary Figs S2D, S3F,G). Cells in the cortex showed higher signal numbers, and 20 RGEN-ISL signals of CentO were observed in two cells in the cortex (Supplementary Fig. S2D). The size of the CentO tracts varies among chromosomes, and there is an approximately 30-fold difference in the number of repeats (Cheng *et al.*, 2002, Table S2). The 3D RGEN-ISL results (Supplementary Fig. S2D) imply that a median of 7–8 pair centromeres, and 10 pair centromeres in the best case, were detected, meaning that the sensitivity of 3D RGEN-ISL is 770 (chromosome 3)–1370 (chromosome 7) copies in the median, and the detection limit is between 460 (chromosome 4) and 660 (chromosome 5) copies. This predicted detection limit is almost the same as the predicted detection limit for telomeres in tomato.

Ten RGEN-ISL signals of centromeric 180-bp repeats were observed in almost all of the meristem cells and cells in the cortex (median=10.0, *n*=10, Supplementary Figs S2E, S3H). GmCent-1 was employed for the detection of soybean centromeres by RGEN-ISL. The repeats showed 22 major and 18 minor signals on 40 soybean centromeres in FISH (Tek *et al.*, 2010). In the 3D RGEN-ISL, signals were observed in meristem cells (median=20.0, *n*=10) and cells in the cortex of the elongation region (median=24.0, *n*=10), and the highest number of signals was 26 in the cortex (Supplementary Figs S2F, S3I, J). The 3D RGEN-ISL could thus detect 22 major and a few minor sites. In common bean, a centromeric tandem repeat, CentPV, which showed eight major and eight minor signals in FISH (Iwata *et al.*, 2013), was used as the target of 3D RGEN-ISL. In the 3D RGEN-ISL, 16 signals were observed in the best cases, and median numbers of signals were 13.5 in meristem cells and 14.0 in cells in the cortex of the elongation region (Supplementary Figs S2G, S3K, L, *n*=10). The sensitivity of the 3D RGEN-ISL was considered to be similar to that of the reported FISH.

**Fig. 3.**
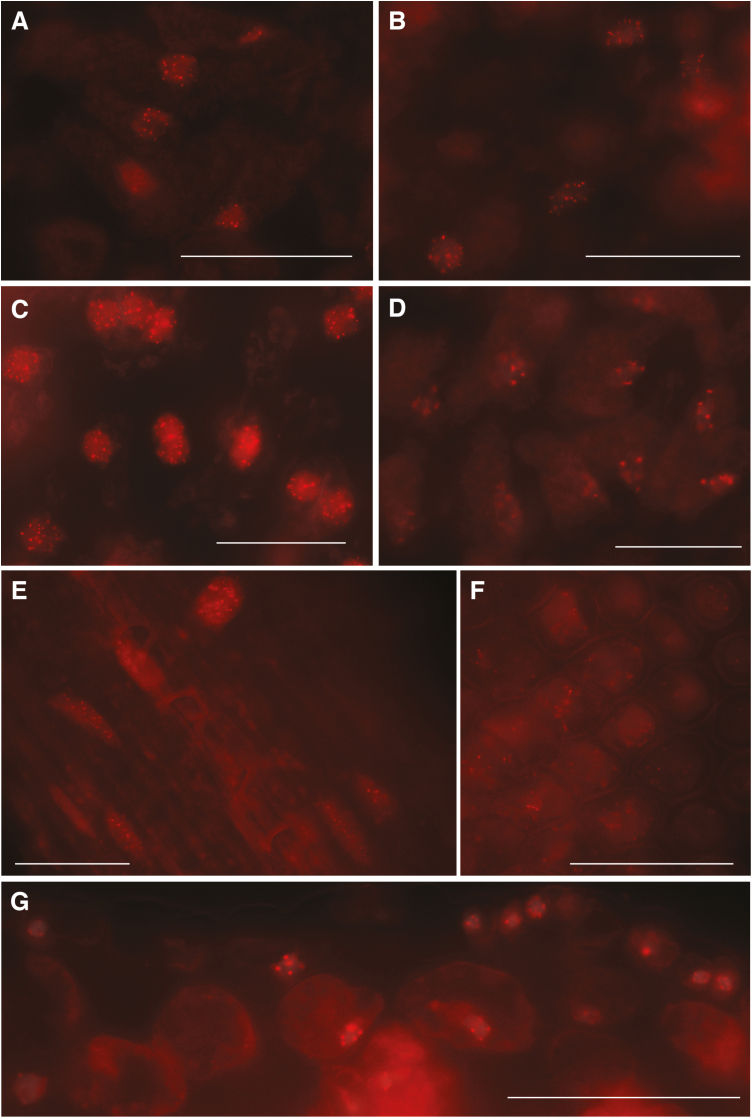
3D RGEN-ISL for leaves, a stem, a petal, and a cotyledon. RGEN-ISL telomere signals in mesophyll regions of sunflower (A), tomato (B), and tobacco (C) leaves, and a region around the xylem of the stem (E) and a parenchyma region of petal (F) of sunflower. RGEN-ISL centromeric DNA signals (180-bp family) in a mesophyll region of leaf (D) and the inner and epidermal area of a cotyledon (G) of Arabidopsis. Scale bar=50 μm.

Next, the 3D RGEN-ISL method was applied to plant tissues other than roots. Since there was a difference in antibody permeability for each plant tissue in immunohistochemistry (Nagaki *et al.*, 2017), the tissue adaptability of 3D RGEN-ISL was examined using leaves, stems, petals, and cotyledons. Clear RGEN-ISL signals were observed in sunflower, tomato, tobacco, and Arabidopsis leaves (Fig. 3A–D; Supplementary Fig. S4A–J). These RGEN-ISL signals were detectable at least in areas 100 μm away from the cutting points in all the cut leaves and petals used, and often in areas more than 1 mm away from the cutting points. In the best case, the RGEN-SIL signals were detectable in all areas of the 5 mm diameter tobacco leaf disk (Supplementary Fig. S5).

In sunflower, the telomere RGEN-ISL signals were observed almost in all cell types in the leaves, but cells in leaves showed fewer signals [median=33.5 (mesophyll in Supplementary Fig. S4A), 0.0 (guard cell in Supplementary Fig. S4A, B), and 44.0 (bundle sheath cell in Supplementary Fig. S4C), *n*=10] than the root cells (Supplementary Fig. S2A). In particular, only small portions of guard cells showed the signals (Supplementary Figs S2A, S4A, B). The difficulty in detecting signals in guard cells may be due to poor entry of RNP into guard cells, because guard cells that were reached by RNP showed similar number of signals as other leaf cells (Supplementary Figs S2A, S4B). Tomato and tobacco leaf cells also showed less signal [median=33.0 (mesophyll), 18.5 (guard cell) and 18.5 (bundle sheath) in tomato, and 47.5 (mesophyll), 47.0 (guard cell) and 51.0 (bundle sheath) in tobacco, *n*=10] than the root cells of the respective species, but in tobacco leaves, unlike sunflower and tomato, the RNP entered all the guard cells, and so guard cells showed similar levels of signal (Supplementary Figs S2B, C, S4D–G).

In Arabidopsis leaf cells, different numbers of centromere signals were observed [median=8.5 (mesophyll), 8.0 (guard cell) and 10.0 (bundle sheath), *n*=10] than in the root cells, but the same number of chromocenters as those signals were observed in the same cells, and they overlapped (Supplementary Figs S2E, S4H–J, S6A–F). This overlap suggests that the divergence in signal number was due to endopolyploidization and centromere association rather than detection failure. In the upper region of the leaf, a small number of centromere signals and centromere associations were observed in palisade mesophyll cells (Supplementary Fig. S6A, B, G). In the middle region of the leaf, 5–9 centromeric signals per cell were observed (Supplementary Fig. S6C, D, H). In the lower region of the leaf, 20 centromere signals were observed in spongy mesophyll cells (Supplementary Fig. S6E, F, I), suggesting that endopolyploidization had occurred in these cells. The association of centromeres that was observed in palisade mesophyll cells was not seen in the spongy mesophyll cells (Supplementary Fig. S6I).

In addition, clear RGEN-ISL signals were observed in the stems and petals of sunflower and the cotyledons of Arabidopsis (Fig. 3E–G). These results imply that the 3D RGEN-ISL method can be applied to many different plant tissues. Since the sunflower stem was sectioned, the number of telomere signals (median=48.5, *n*=10) was similar to that of sunflower root (Supplementary Figs S2A, S4K). On the other hand, the sunflower petals were only cleared, so they showed the same level of signals (median=43.5, *n*=10) as sunflower leaves (Supplementary Figs S2A, S4L). In addition, cells in sectioned Arabidopsis cotyledons showed a similar level of centromere signal [median=10.0 (inner cell) and 9.0 (epidermis), *n*=10] as Arabidopsis roots (Supplementary Figs S2E, S4M).

**Fig. 4.**
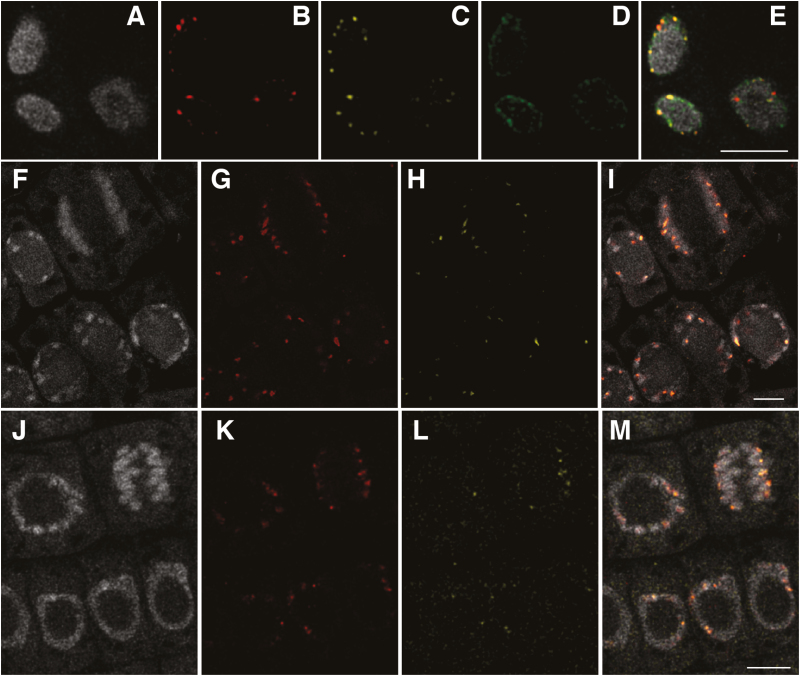
High-resolution confocal analyses of 3D RGEN-ISL and immunohistochemistry signals. DAPI-stained nuclei of rice (A), soybean (F), and common bean (J). RGEN-ISL centromeric DNA signals in rice (CentO) (B), soybean (G71) (G), and common bean (CentPv) (K). Immunohistochemistry signals of CENH3 in rice (C), soybean (H), and common bean (L), and K9 dimethylated histone H3 (D). (E) Merged image of A–D; (I) merged image of F–H; (M) merged image of J–L. Scale bar=5 μm.

Furthermore, we checked the compatibility of the 3D RGEN-ISL method and immunohistochemistry. Colocalization of the centromeric RGEN-ISL signals and signals of kinetochore proteins was confirmed in rice, soybean, and common bean by high-resolution analyses using a confocal microscope and software for three-dimensional deconvolution analyses (Fig. 4A–C, E–M). In addition to the kinetochore proteins, signals of histone modification (K9 dimethylated histone H3) were also visualized in rice (Fig. 4D, E). These results imply that the 3D RGEN-ISL method is applicable for colocalization analyses of target DNA sequences and chromosomal proteins. Surprisingly, chromatin structures of chromosomes were visible in the interphase nuclei of rice by high-resolution analyses (Fig. 4A, E; Supplementary Video S1). This result demonstrated that chromatin structures were maintained even after 3D RGEN-ISL and indicates the possibility of high-resolution chromosome mapping using 3D RGEN-ISL.

In this research, we developed a universal method to visualize repetitive DNA sequences in various plant tissues (Figs 1–3; Supplementary Figs S2–S4). The method is compatible with immunohistochemistry of tissues that require fixation (Fig. 4). Furthermore, the signals obtained by the method were sufficiently strong to use for high-resolution analyses (Fig. 4). Since this method can visualize target DNA signals on chromosomes in tissues, it could be applied to analyses of the chromosome territory of different types of cells in tissues, the dynamics of chromosomes from each parent in hybrids, including chromosome elimination, and chromosome doubling systems in tissues. In mammals, a single locus was detected by a CRISPR/Cas9 system using 57 tiled sgRNAs (Deng *et al.*, 2015), meaning that approximately 60 localized fluorescent molecules are sufficient to visualize a single locus. If we could design such tiled crRNAs, the visualization of a single locus using the 3D RGEN-ISL method might be possible in plant tissues. Such a high-sensitivity system would allow further applications of this method. In particular, combinations of the 3D RGEN-ISL method and immunohistochemistry for epigenetic modifications will enable the high-resolution mapping of chromatin modifications in individual cells in plant tissues, and these analyses will shed light on coordination in the regulation of gene expression of individual cells in plant tissues.

## Supplementary data

Supplementary data are available at *JXB* online.

**Table S1.** List of crRNA sequences used for this research.

**Table S2.** Distribution of CentO repeat on rice cv. Nipponbare centromeres.

**Fig. S1.** Effect of reaction temperature on RGEN-ISL.

**Fig. S2.** RGEN-ISL signals observed in cells of various tissues and species.

**Fig. S3.** Full focus processed images of 3D RGEN-ISL using root sections of various species.

**Fig. S4.** Full focus processed images of 3D RGEN-ISL using leaf, stem and cotyledon sections of various species.

**Fig. S5.** 3D RGEN-ISL for a 5 mm diameter tobacco leaf disk.

**Fig. S6.** 3D RGEN-ISL for an Arabidopsis leaf.

**Video S1.** High-resolution confocal analyses of the 3D RGEN-ISL and immunohistochemistry signals in rice.

erz534_suppl_Supplementary_Tables_S1-S2_Figures_S1-S6

erz534_suppl_Supplementary_Video_S1

## Abbreviations:

3D: three-dimensional
Cas9: CRISPR-associated caspase 9
CENH3: centromere-specific histone H3 variant
CRISPR: clustered regularly interspaced short palindromic repeats
FISH: fluorescent *in situ* hybridization
PFA: paraformaldehyde
RGEN-ISL: RNA-guided endonuclease–*in situ* labeling
RNP: ribonucleoprotein
WLL: white light laser

## References

[CIT0001] Chen B, Gilbert LA, Cimini BA, et al 2013 Dynamic imaging of genomic loci in living human cells by an optimized CRISPR/Cas system. Cell 155, 1479–1491. 24360272 10.1016/j.cell.2013.12.001 PMC3918502

[CIT0002] Cheng Z, Dong F, Langdon T, Ouyang S, Buell CR, Gu M, Blattner FR, Jiang J 2002 Functional rice centromeres are marked by a satellite repeat and a centromere-specific retrotransposon. The Plant Cell 14, 1691–1704. 12172016 10.1105/tpc.003079 PMC151459

[CIT0003] Deng W, Shi X, Tjian R, Lionnet T, Singer RH 2015 CASFISH: CRISPR/Cas9-mediated in situ labeling of genomic loci in fixed cells. Proceedings of the National Academy of Sciences, USA 112, 11870–11875. 10.1073/pnas.1515692112 PMC4586837 26324940

[CIT0004] Dreissig S, Schiml S, Schindele P, Weiss O, Rutten T, Schubert V, Gladilin E, Mette MF, Puchta H, Houben A 2017 Live-cell CRISPR imaging in plants reveals dynamic telomere movements. The Plant Journal 91, 565–573. 28509419 10.1111/tpj.13601 PMC5599988

[CIT0005] Fujimoto S, Sugano SS, Kuwata K, Osakabe K, Matsunaga S 2016 Visualization of specific repetitive genomic sequences with fluorescent TALEs in *Arabidopsis thaliana*. Journal of Experimental Botany 67, 6101–6110. 27811079 10.1093/jxb/erw371 PMC5100022

[CIT0006] Ganal MW, Lapitan NL, Tanksley SD 1991 Macrostructure of the tomato telomeres. The Plant Cell 3, 87–94. 1668615 10.1105/tpc.3.1.87 PMC159981

[CIT0007] Hübner MR, Eckersley-Maslin MA, Spector DL 2013 Chromatin organization and transcriptional regulation. Current Opinion in Genetics & Development 23, 89–95. 23270812 10.1016/j.gde.2012.11.006 PMC3612554

[CIT0008] Ishii T, Schubert V, Khosravi S, Dreissig S, Metje-Sprink J, Sprink T, Fuchs J, Meister A, Houben A 2019 RNA-guided endonuclease – *in situ* labelling (RGEN-ISL): a fast CRISPR/Cas9-based method to label genomic sequences in various species. New Phytologist 222, 1652–1661. 30847946 10.1111/nph.15720 PMC6593734

[CIT0009] Iwata A, Tek AL, Richard MM, et al 2013 Identification and characterization of functional centromeres of the common bean. The Plant Journal 76, 47–60. 23795942 10.1111/tpj.12269

[CIT0010] Kato N, Lam E 2001 Detection of chromosomes tagged with green fluorescent protein in live *Arabidopsis thaliana* plants. Genome Biology 2, RESEARCH0045.1. 11737944 10.1186/gb-2001-2-11-research0045 PMC60307

[CIT0011] Kozubek S, Lukásová E, Amrichová J, Kozubek M, Lisková A, Slotová J 2000 Influence of cell fixation on chromatin topography. Analytical Biochemistry 282, 29–38. 10860496 10.1006/abio.2000.4538

[CIT0012] Kurihara D, Mizuta Y, Sato Y, Higashiyama T 2015 ClearSee: a rapid optical clearing reagent for whole-plant fluorescence imaging. Development 142, 4168–4179. 26493404 10.1242/dev.127613 PMC4712841

[CIT0013] Lindhout BI, Fransz P, Tessadori F, Meckel T, Hooykaas PJ, van der Zaal BJ 2007 Live cell imaging of repetitive DNA sequences via GFP-tagged polydactyl zinc finger proteins. Nucleic Acids Research 35, e107. 17704126 10.1093/nar/gkm618 PMC2018617

[CIT0014] Ma H, Reyes-Gutierrez P, Pederson T 2013 Visualization of repetitive DNA sequences in human chromosomes with transcription activator-like effectors. Proceedings of the National Academy of Sciences, USA 110, 21048–21053. 10.1073/pnas.1319097110 PMC3876203 24324157

[CIT0015] Matzke AJ, Huettel B, van der Winden J, Matzke M 2005 Use of two-color fluorescence-tagged transgenes to study interphase chromosomes in living plants. Plant Physiology 139, 1586–1596. 16339805 10.1104/pp.105.071068 PMC1310544

[CIT0016] Nagaki K, Cheng Z, Ouyang S, Talbert PB, Kim M, Jones KM, Henikoff S, Buell CR, Jiang J 2004 Sequencing of a rice centromere uncovers active genes. Nature Genetics 36, 138–145. 14716315 10.1038/ng1289

[CIT0017] Nagaki K, Tanaka K, Yamaji N, Kobayashi H, Murata M 2015 Sunflower centromeres consist of a centromere-specific LINE and a chromosome-specific tandem repeat. Frontiers in Plant Science 6, 912. 26583020 10.3389/fpls.2015.00912 PMC4628103

[CIT0018] Nagaki K, Yamaji N, Murata M 2017 ePro-ClearSee: a simple immunohistochemical method that does not require sectioning of plant samples. Scientific Reports 6, srep42203. 10.1038/srep42203 PMC5297246 28176832

[CIT0019] Naito Y, Hino K, Bono H, Ui-Tei K 2015 CRISPRdirect: software for designing CRISPR/Cas guide RNA with reduced off-target sites. Bioinformatics 31, 1120–1123. 25414360 10.1093/bioinformatics/btu743 PMC4382898

[CIT0020] Tek AL, Kashihara K, Murata M, Nagaki K 2010 Functional centromeres in soybean include two distinct tandem repeats and a retrotransposon. Chromosome Research 18, 337–347. 20204495 10.1007/s10577-010-9119-x

[CIT0021] Xiang G, Zhang X, An C, Cheng C, Wang H 2017 Temperature effect on CRISPR-Cas9 mediated genome editing. Journal of Genetics and Genomics 44, 199–205. 28412228 10.1016/j.jgg.2017.03.004

