# Supplementary material for: Decrosslinking enables visualization of RNA-guided endonuclease–in situ labeling signals for DNA sequences in plant tissues
Source: J Exp Bot. 2019 Nov 30;71(6):1792–800. doi: 10.1093/jxb/erz534 (PMC7094073; doi:10.1093/jxb/erz534)
Supplement: erz534_suppl_Supplementary_Tables_S1-S2_Figures_S1-S6 [file erz534_suppl_supplementary_tables_s1-s2_figures_s1-s6.pdf]

Table S1. List of crRNA sequences used for this research

Name	Sequence
Arabidopsis-type telomere	**GGGUUUAGGGUUUAGGGUUU**GUUUUAGAGCUAUGCU
Arabidopsis centromere	**UUGAGAAGCAAGAAGAAGGU**GUUUUAGAGCUAUGCU
Rice centromere (CentO)	**UUGGACAUAUUGGAGUGUAU**GUUUUAGAGCUAUGCU
Soybean centromere (GmCent-1: G71)	**AGACGCUCGAAAUUGAACAA**GUUUUAGAGCUAUGCU
Common bean centromere (CentPv)	**AAUACUUCUCUUACAAUCUA**GUUUUAGAGCUAUGCU

The sequences indicated in bold are target-specific regions of crRNA.

Table S2. Distribution of CentO repeat on Rice cv. Nipponbare centromeres.

Chromosome	Relative Intensity of FISH Signal (Cheng *et al.*, 2002)	[a]Size of CentO Tract (kb)	[b]Copy Number of CentO	CentO copy number ranking
1	22.0	904	6,024	2
2	11.1	457	3,045	4
3	2.8	116	774	8
4	1.7	69	459	11
5	2.4	98	656	10
6	12.7	520	3,469	3
7	5.0	205	1,367	7
8	1.0	41	273	12
9	9.6	392	2,616	5
10	7.3	300	2,001	6
11	29.5	1,209	8,058	1
12	2.4	100	664	9

[a]The CentO contents in the each chromosome were calculated by the relative intensity of FISH signals and a reported CentO content in a centromere of Chromosome 8 (Nagaki *et al.*, 2004).

[b]The numbers of CentO were calculated by the CentO contents and an average repetitive unit size of CentO (155 bp).

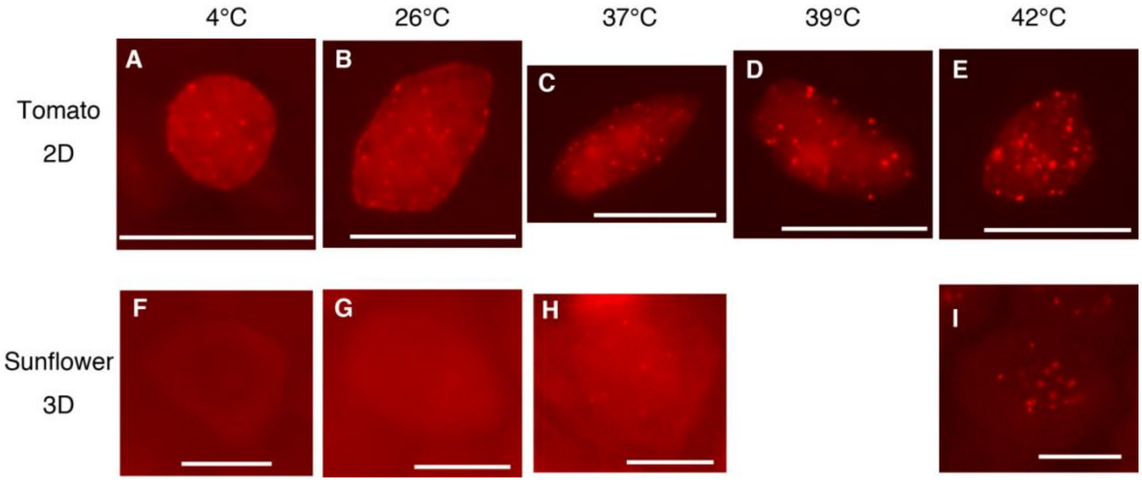

Figure S1. Effect of reaction temperature on 2D and 3D RGEN-ISL

RGEN-ISL telomere signals on released tomato nuclei (A-E) and nuclei in sunflower root sections (F-I). The reaction temperatures of RGEN-ISL are shown in the upper part. Scale bar, 10 µm.

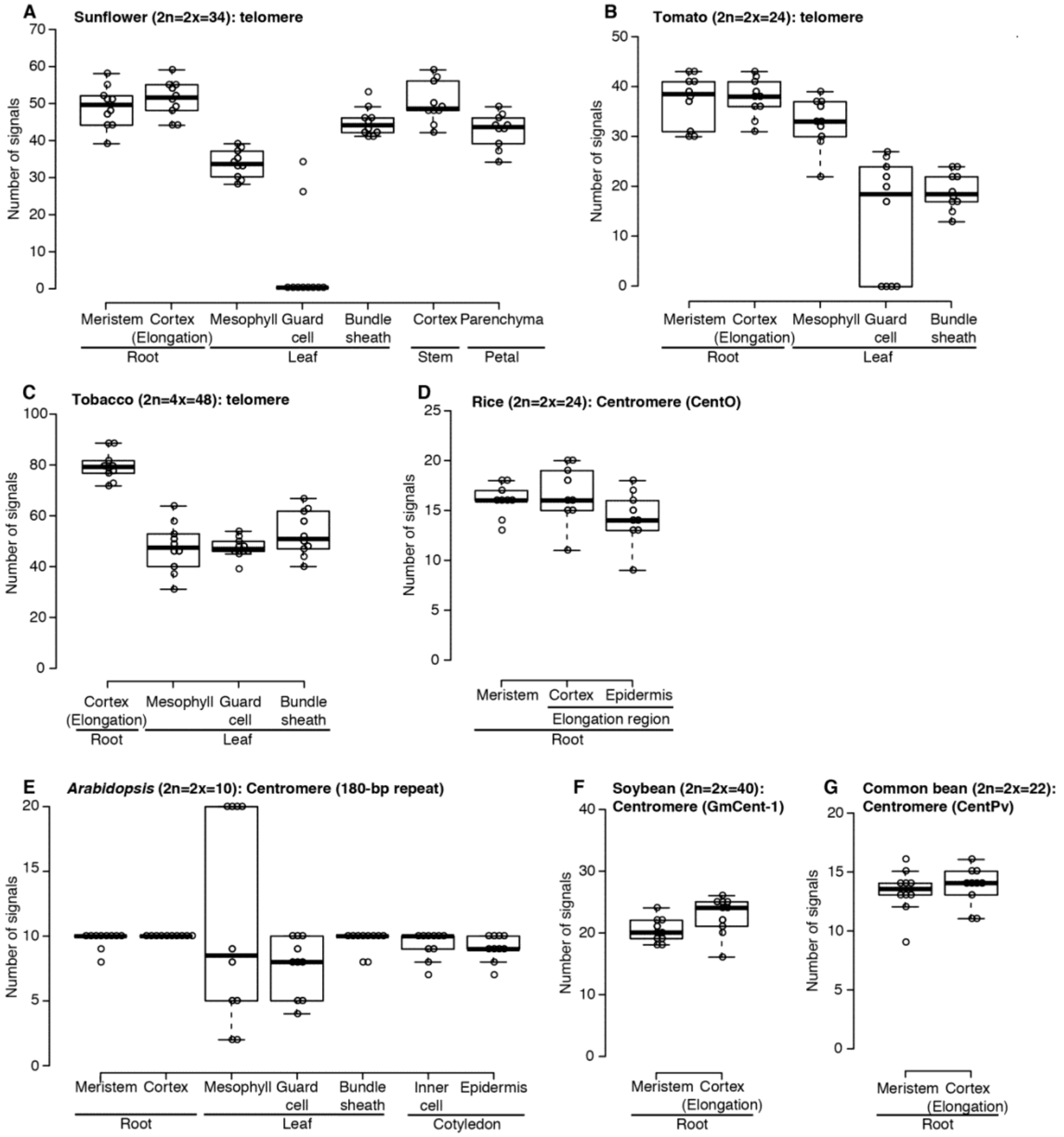

Figure S2. RGEN-ISL signals observed in cells of various tissues and species.

In all cell types, RGEN-ISL signals in ten nuclei were counted using sets of Z-stack images. Center lines show the medians; box limits indicate the 25th and 75th percentiles; whiskers extend 1.5 times the interquartile range from the 25th and 75th percentiles, outliers are represented by dots; data points are plotted as open circles.

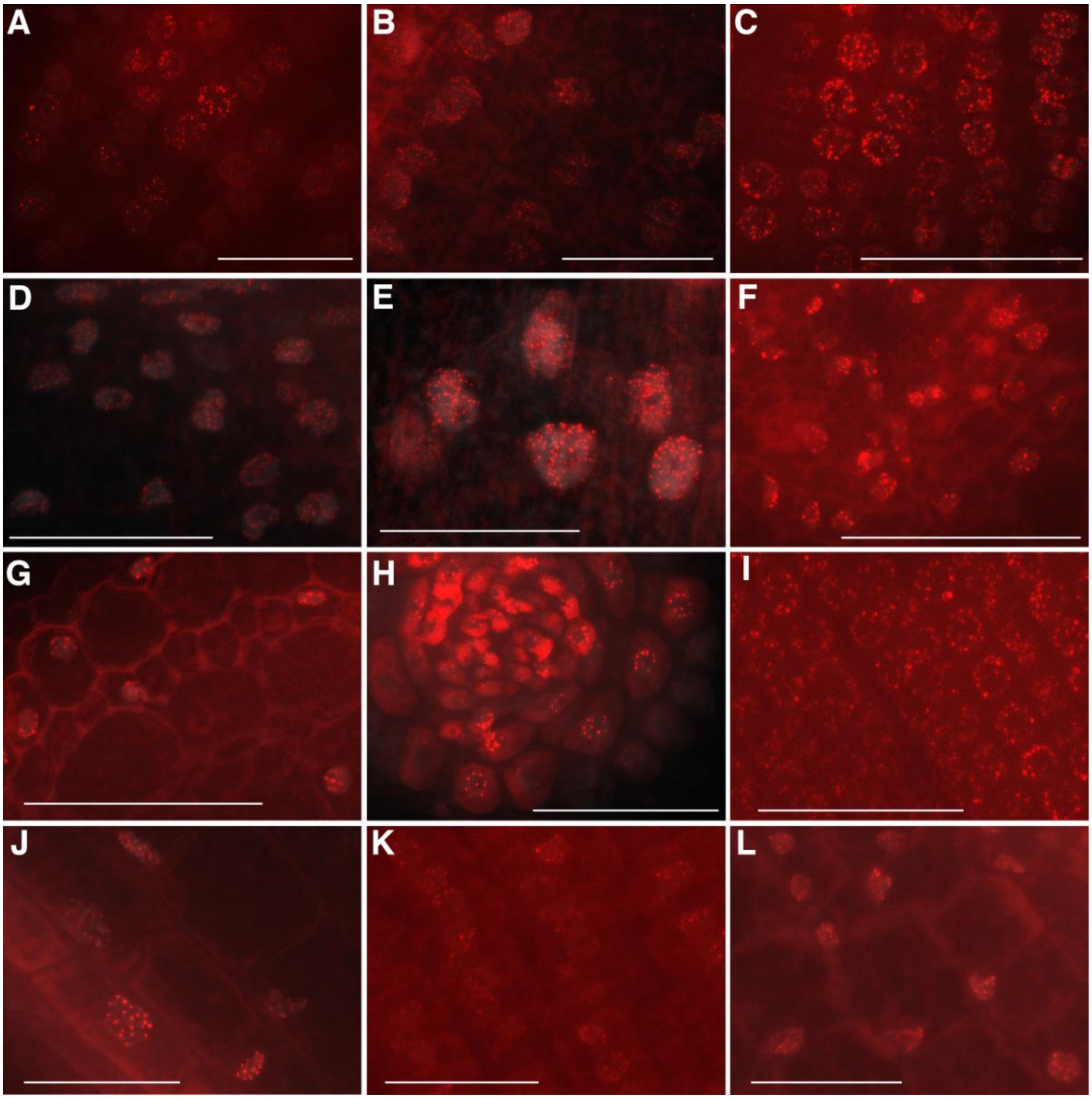

Figure S3. Full focus processed images of 3D RGEN-ISL using root sections of various species.

Telomere RGEN-ISL signals in meristem (A) and elongation (B) regions of sunflower. The telomere signals in meristem (C) and elongation (D) regions of tomato. The telomere signals in an elongation region (E) of tobacco. CentO (centromeric) RGEN-ISL signals in meristem (F) and elongation (G) regions of rice. RGEN-ISL signals of 180-bp repeats (centromeric) in a meristem region (H) of *Arabidopsis*. GmCent-1 (centromeric) RGEN-ISL signals in meristem (I) and elongation (J) regions of soybean. CentPv (centromeric) RGEN-ISL signals in meristem (K) and elongation (L) regions of common bean. DAPI-stained nuclei (gray), RGEN-ISL signals (red) were visualized. Scale bar, 50 μm.

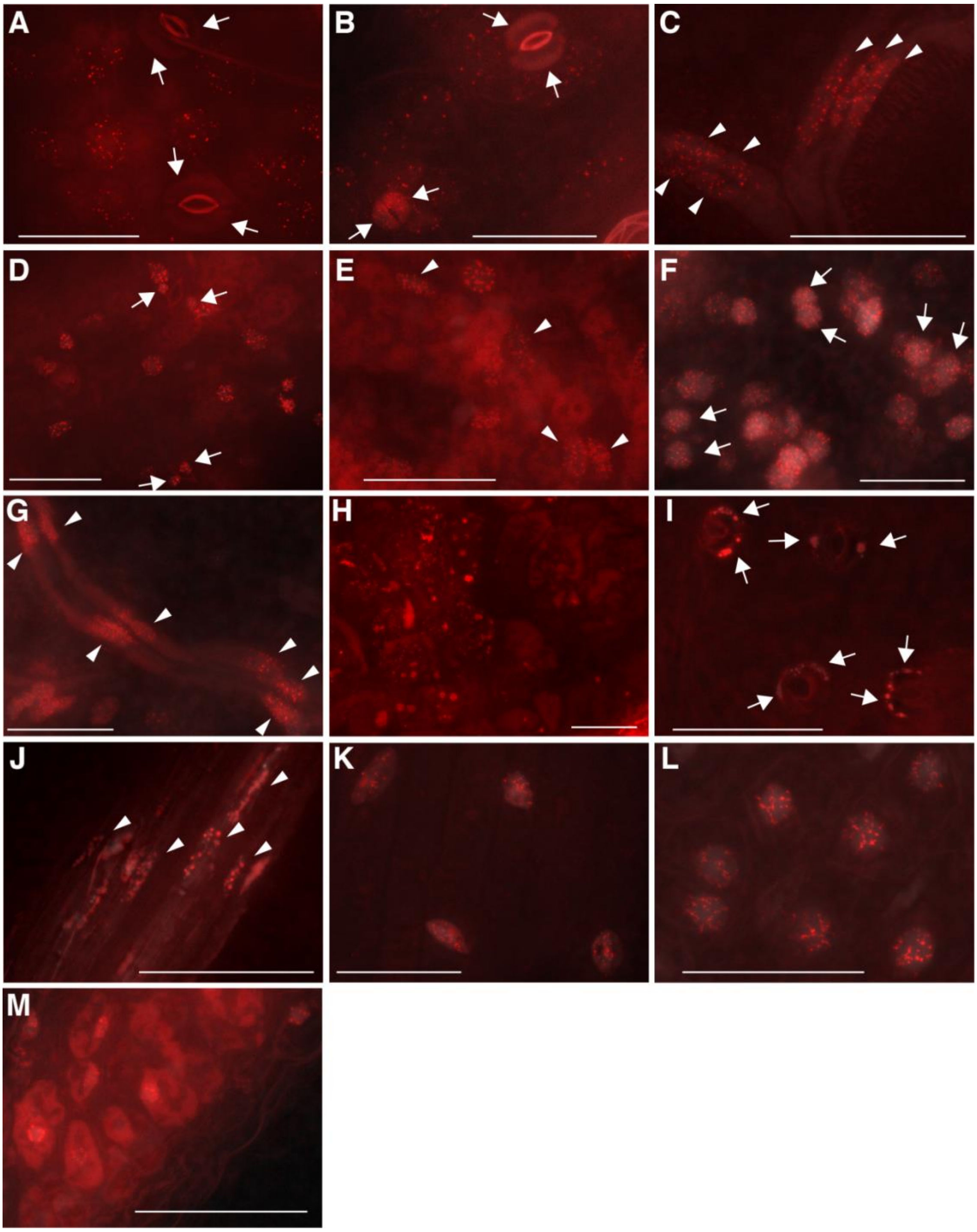

Figure S4. Full focus processed images of 3D RGEN-ISL using leaf, stem and cotyledon sections of various species.

Telomere RGEN-ISL signals in leaves (A-C), a stem (K) and a petal (L) of sunflower. The telomere signals in leaves of tomato (D and E). The telomere signals in leaves of tobacco (F and G). RGEN-ISL signals of 180-bp repeats (centromeric) in leaves (H-J) and a cotyledon (M) of *Arabidopsis*. DAPI-stained nuclei (gray), RGEN-ISL signals (red) were visualized. Arrows and arrowheads indicate guard cells and bundle sheath cells, respectively. Scale bar, 50 μm.

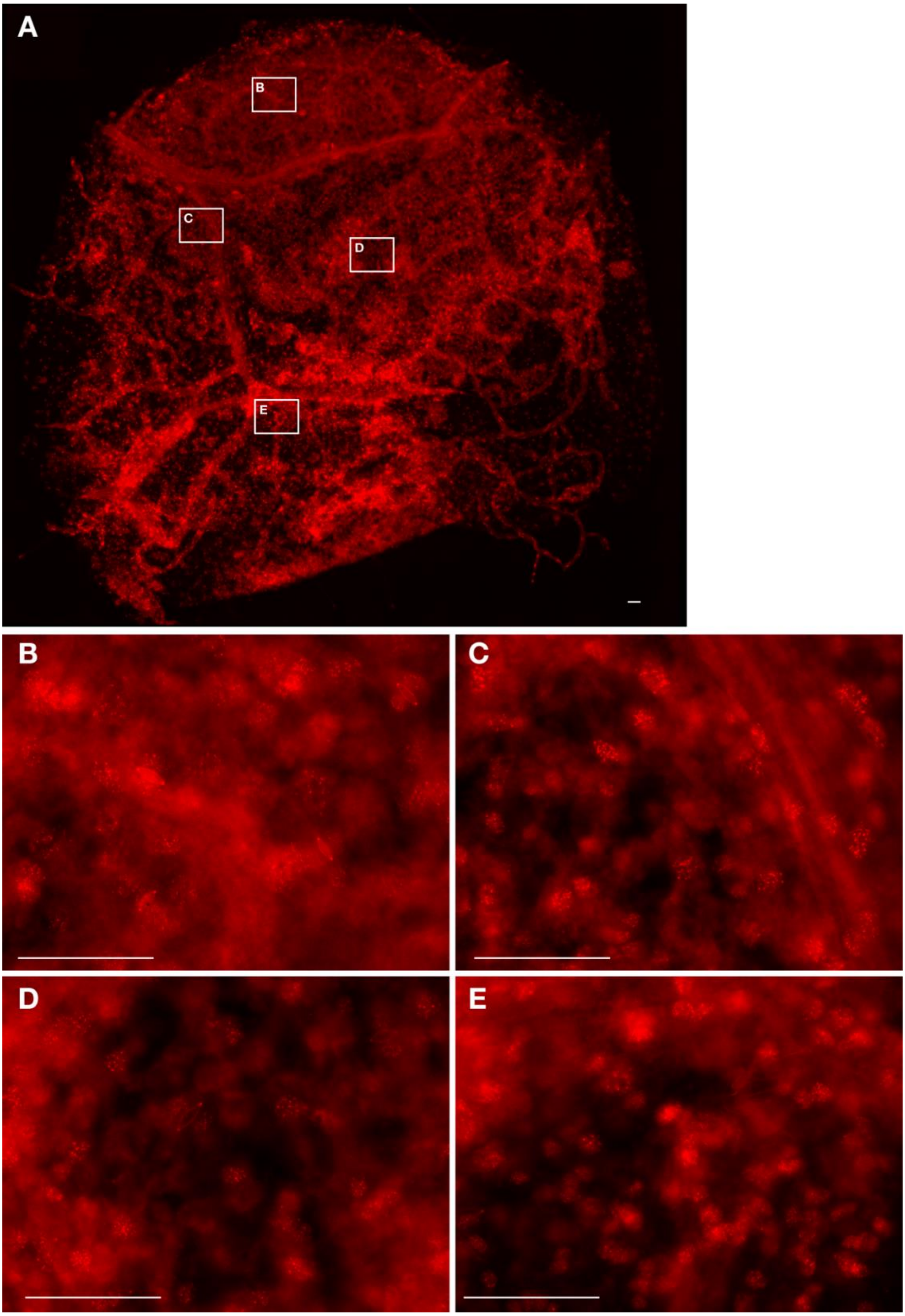

Figure S5. 3D RGEN-ISL for a 5 mm diameter tobacco leaf disk

RGEN-ISL telomere signals in a tobacco leaf disk. Panels B-E show enlarged images of the boxes in Panel A. RGEN-ISL signals (red) were visualized. Scale bar, 100 µm.

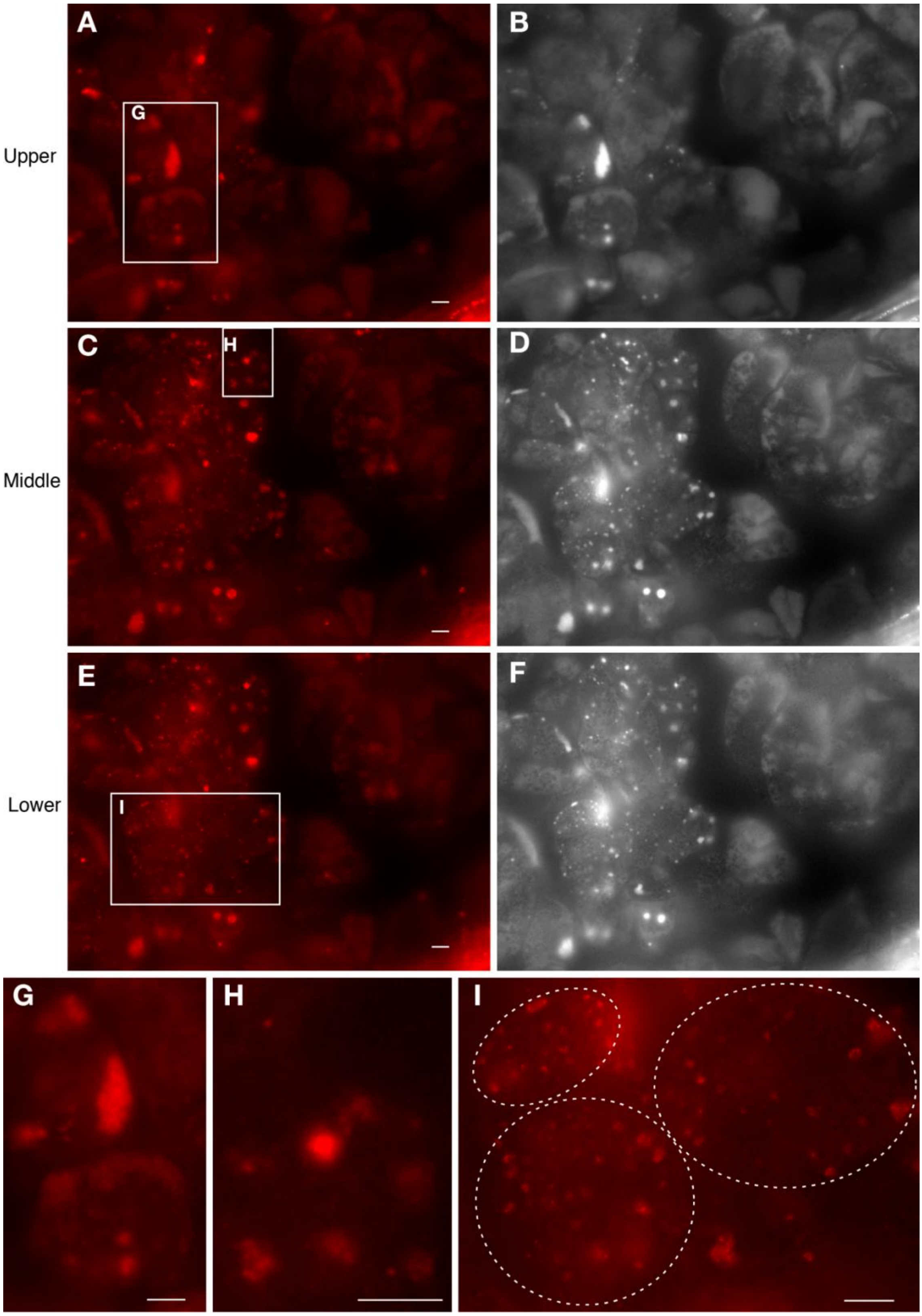

Figure S6. 3D RGEN-ISL for an *Arabidopsis* leaf

RGEN-ISL telomere and DAPI signals in the upper (A and B), the middle (C and D), the lower regions (E and F) of an *Arabidopsis* leaf. Panel G, H and I show enlarged images of the boxes in Panel A, C and E, respectively. The areas surrounded by the

dotted line in panel I represents spongy mesophyll cells. Scale bar, 10 μm.

Video S1. High-resolution confocal analyses of the 3D RGEN-ISL and immunohistochemistry signals in rice

DAPI-stained nuclei (gray), RGEN-ISL centromeric DNA (CentO) signals in rice (red), immunohistochemistry signals of CENH3 (yellow), and K9 dimethylated histone H3 (green) were visualized in this section.
